# Supplementary material for: Raman Spectroscopy Spectral Fingerprints of Biomarkers of Traumatic Brain Injury
Source: Cells. 2023 Nov 8;12(22):2589. doi: 10.3390/cells12222589 (PMC10670390; doi:10.3390/cells12222589)
Supplement: Supplementary file 1 [file cells-12-02589-s001.zip › cells-2685012-supplementary.pdf]

- SUPPORTING INFORMATION -

Raman Spectroscopy Spectral Fingerprints of Biomarkers of Traumatic Brain Injury

Georgia Harris[1], Clarissa A. Stickland[1], Matthias Lim[1], Pola Goldberg Oppenheimer[1,2,*]

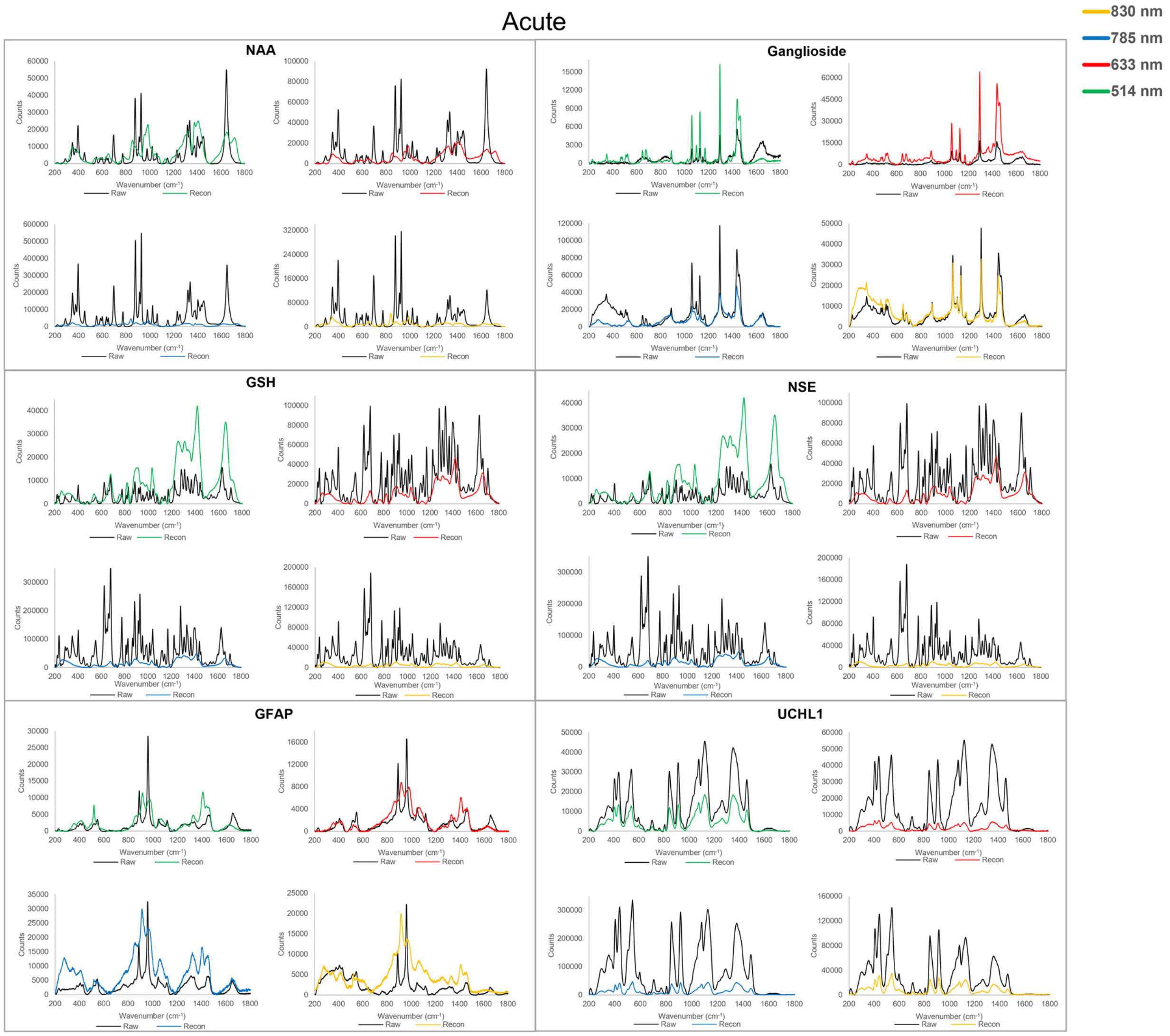

Figure S1. Spectra of raw and reconstituted acute TBI biomarkers of the NAA (top left), ganglioside (top right), GSH (middle left), NSE (middle right), GFAP (bottom left) and UCHL1 (bottom right), using all four laser excitation wavelengths 514 (green), 633 (red), 785 (blue) and 830 nm (yellow).

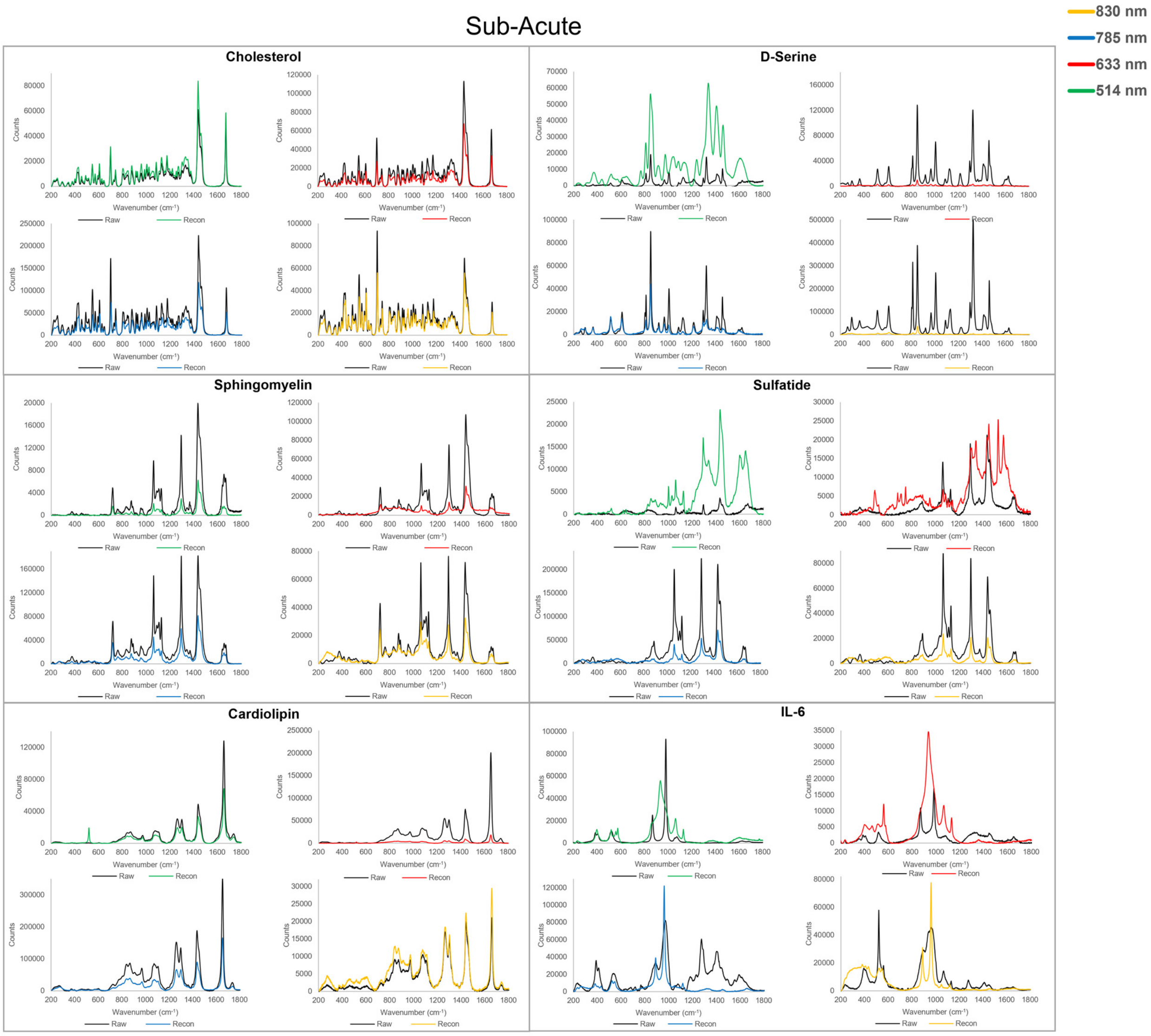

Figure S2. Spectra of raw and reconstituted sub-acute TBI biomarkers: cholesterol (top left), D-serine (top right), sphingomyelin (middle left), sulfatide (middle right), cardiolipin (bottom left) and IL-6 (bottom right), using all four laser excitation wavelengths 514 (green), 633 (red), 785 (blue) and 830 nm (yellow).

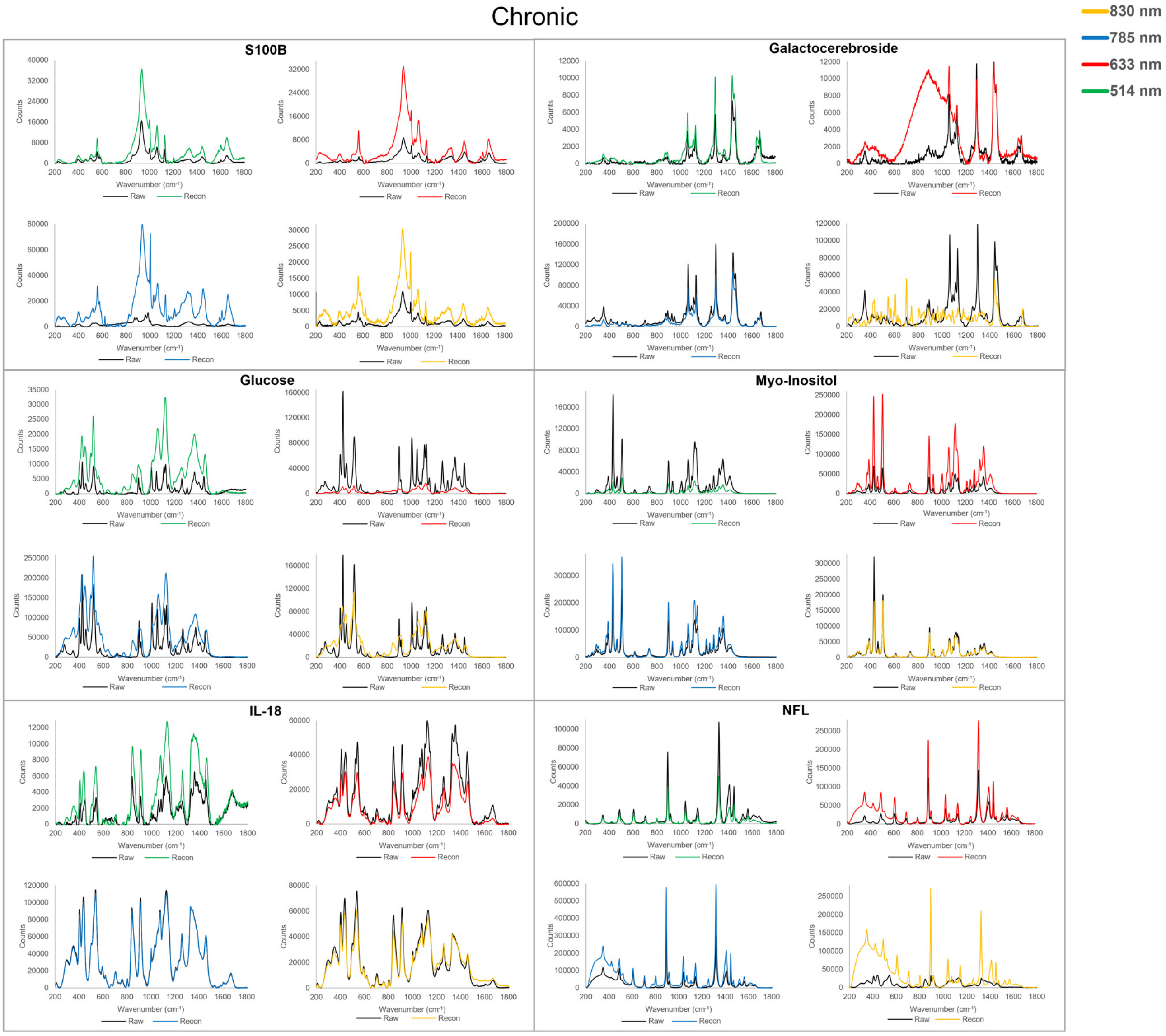

Figure S3. Spectra of raw and reconstituted Chronic TBI biomarkers: S100B (top left), galactocerebroside (top right), glucose (middle left), myo-inositol (middle right), IL-18 (bottom left) and NFL (bottom right), using all four laser excitation wavelengths 514 (green), 633 (red), 785 (blue) and 830 nm (yellow).
